# A new species of *Raveniola* Zonstein, 1987 (Araneae, Nemesiidae) from Fujian, China

**DOI:** 10.3897/BDJ.13.e142264

**Published:** 2025-01-03

**Authors:** Guchun Zhou, Jian Lu, Muqiushi Cui, Jiasheng Xu

**Affiliations:** 1 School of Life Sciences, National Navel Orange Engineering Research Center, Gannan Normal University, Ganzhou, China School of Life Sciences, National Navel Orange Engineering Research Center, Gannan Normal University Ganzhou China; 2 Administration of Jiangxi Qiyunshan National Nature Reserve, Ganzhou, China Administration of Jiangxi Qiyunshan National Nature Reserve Ganzhou China

**Keywords:** Asia, biodiversity, morphology, Mygalomorphae, taxonomy

## Abstract

**Background:**

The genus *Raveniola* Zonstein, 1987 comprises 66 species, distributed across regions from East Asia to the Caucasus, with about 20 species recorded from China. According to Zonstein et al. (2018) and Zonstein (2024), members of *Raveniola* can be identified by the presence of two to three retroventral megaspines arranged sequentially on tibia I in males and paired spermathecae in females, each bearing two-branched heads or a lateral diverticulum.

**New information:**

A new mygalomorph species, *Raveniolafuzhouensis* Zhou, sp. nov., is described from Fujian Province, China. Detailed description, diagnosis, illustrations and a distribution map of the new species are provided.

## Introduction

The family Nemesiidae comprises 182 extant species across 10 genera, along with four fossil species in four genera, distributed worldwide, out of which, 26 species in three genera have been recorded in China (World Spider Catalog 2024). *Raveniola* Zonstein, 1987 species may rely on ecological opportunities rather than actively digging their own burrows. While *Raveniola* does not produce large quantities of silk in the burrow, as many other mygalomorphs do, this does not mean they completely avoid using silk. In fact, they use a small amount of fine, thin silk to line the interior of their burrows. Although they do not spin complex webs within these tunnels, *Raveniola* remains active at night, waiting to ambush passing arthropods as they traverse the area (Zonstein 2024). *Raveniola* species are found across a wide range of altitude gradients, a pattern observed not only in Chinese species, but also in those from Central Asia (Andreeva 1975, Zonstein 2024). These spiders inhabit a variety of environments, including underground caves (Lin et al. 2022, Lin and Li 2022), alpine meadows, forests and other diverse ecosystems. Within these habitats, they occupy a range of microhabitats such as slopes, scree piles, deep fissures in large rocks and abandoned vertebrate nests, demonstrating their remarkable adaptability to different ecological niches. In China, a total of 20 species of *Raveniola* have been documented (World Spider Catalog 2024). The majority of these species (16) are found in the south-western regions, including Yunnan (nine species), Guangxi (five species), Sichuan (one species) and Xizang (one species) (Zonstein and Marusik 2012, Zonstein et al. 2018, Lin and Li 2020, Yu and Zhang 2021, Lin et al. 2024). Three species are recorded from the central and northern regions, with one species each from Hunan, Hubei and Hebei. Additionally, a single species, *Raveniolagracilis* (Li and Zonstein 2015), is known from the south-eastern coastal region of Zhejiang. *Raveniola* spiders are distributed across a range of altitudes in China, from low-altitude coastal cities in Zhejiang to the high-altitude regions of Yunnan and Tibet, demonstrating the genus' strong adaptability across diverse environments (Zonstein and Marusik 2012, Zonstein et al. 2018, Yu and Zhang 2021).

Fuzhou City is located in the southeast coast of China, belonging to the subtropical marine monsoon climate, warm and humid, evergreen, abundant rainfall, the average annual precipitation being 900 ~ 2100 mm, the average annual temperature 20 ~ 25°C. Fuzhou National Forest Park, situated in northern Fuzhou City is connected to Fuzhou's northern peak. The Park's terrain is primarily hilly and features a mix of artificial and natural secondary forests, with complex vegetation types and predominantly thin red and humus soil layers (Wang et al. 2023). During a recent spider survey, a new *Raveniola* species was discovered, highlighting the need for more comprehensive scientific studies on Arthropoda in the region. The discovery of the second *Raveniola* in the southeast coastal region of China and is the first report of *Raveniola* in Fujian Province.

## Materials and methods

Specimens were collected by handpicking and were kept in 95% ethanol. After dissection, epigyne was cleared by trypsin enzyme solution before examination and photography. Specimens were examined, measured with a Leica MZ6 stereomicroscope. Photos were taken with a Kuy Nice CCD mounted on an Olympus BX41 and stacked with Helicon Focus software (v.3.10) (Khmelik et al. 2005). The map is from SMSS (Standard Map Service System 2024) and then edited using Adobe Photoshop 2021 Extended (Fig. 8). Leg segments were measured on the dorsal side. Leg measurements are given in the following order: total length (femur, patella, tibia, metatarsus, tarsus). All measurements are given in millimetres (mm). The specimens are deposited in the Taxidermy Museum of Gannan Normal University, Ganzhou City, China (GNNU).

Terminology and taxonomic descriptions follow Li and Zonstein (2015) and Zonstein (2024). Abbreviations: **ALE** = anterior lateral eyes, **AME** = anterior median eyes, **CL** = carapace length, **CW** = carapace width, **d** = dorsal, **IS** = inner spermathecal branch, **ld** = lateral diverticulum, **M** = megaspine, **MIT** = male intercheliceral tumescence, **OS** = outer spermathecal branch, **p** = prolateral, **Pb** = psembolus, **pd** = prodorsal, **PLE** = posterior lateral eyes, **PLS** = posterior lateral spinnerets, **PME** = posterior median eyes, **PMS** = posterior median spinnerets, **PTC** = paired tarsal claws, **pv** = proventral, **sb** = spermathecal base, **SD** = sperm duct, **SL** = sternum length, **st** = spermathecal trunk, **SW** = sternum width, **r** = retrolateral, **rd** = retrodorsal, **rv** = retroventral, **TL** = total body length, **v** = ventral.

## Taxon treatments

### Raveniola fuzhouensis

Zhou sp. nov.

ABBE125B-0B6D-5463-AEDC-869AC2CD1E60

B2B48077-4D5F-4936-99C6-7C605BF49ED6

#### Materials

- **Type status:** Holotype. **Occurrence:** recordedBy: Guchun Zhou, Yuanrui Wu; individualCount: 1; sex: male; lifeStage: adult; occurrenceID: 49951440-942C-5D88-8422-2B1E6C81FA1E; **Taxon:** kingdom: Animalia; phylum: Arthropoda; class: Arachnida; order: Araneae; family: Nemesiidae; genus: Raveniola; **Location:** country: China; stateProvince: Fujian; county: Jin'an District; locality: Fuzhou City, Fuzhou National Forest Park; verbatimElevation: 92.2; verbatimLatitude: 26°9′36.7″N; verbatimLongitude: 119°16′50.4″E; **Event:** samplingProtocol: by hand; year: 2024; month: 4; day: 10; **Record Level:** institutionCode: FJFZ-24-19-03
- **Type status:** Paratype. **Occurrence:** recordedBy: Zhou Gu-chun, Wu Yuanrui; individualCount: 1; sex: 1 female; lifeStage: adult (raised to 2024-5-26 mature); occurrenceID: A3A26484-542E-5F5C-9220-B4281F26304F; **Taxon:** kingdom: Animalia; phylum: Arthropoda; class:  Arachnida; order: Araneae; family: Nemesiidae; genus: Raveniola; **Location:** country: China; stateProvince: Fujian; county: Jin'an District; locality: Fuzhou City, Fuzhou National Forest Park; verbatimElevation: 92.2; verbatimLatitude: 26°9′36.7″N; verbatimLongitude: 119°16′50.4″E; **Event:** samplingProtocol: by hand; year: 2024; month: 4; day: 10; **Record Level:** institutionCode: FJFZ-24-19-04
- **Type status:** Paratype. **Occurrence:** recordedBy: Zhou Guchun; individualCount: 2; sex: 2 males; lifeStage: adult; occurrenceID: D5E9D03B-81A9-505A-86C6-4995287AAEB5; **Taxon:** kingdom: Animalia; phylum: Arthropoda; class:  Arachnida; order: Araneae; family: Nemesiidae; genus: Raveniola; **Location:** country: China; stateProvince: Fujian; county: Jin'an District; locality: Fuzhou City, Fuzhou National Forest Park; verbatimElevation: 193.5; verbatimLatitude: 26°10′27.3″N; verbatimLongitude: 119°16′25.8″E; **Event:** samplingProtocol: by hand; year: 2024; month: 1; day: 25; **Record Level:** institutionCode: FJFZ-24-09-04
- **Type status:** Paratype. **Occurrence:** recordedBy: Zhou Guchun; individualCount: 4; sex: 4 males; lifeStage: adult; occurrenceID: D8EF4351-90D0-5F0D-80A2-0F6C66B5BA21; **Taxon:** kingdom: Animalia; phylum: Animalia; class: Animalia; order: Animalia; family: Nemesiidae; genus: Raveniola; **Location:** country: China; stateProvince: Fujian; county: Jin'an District; locality: Fuzhou City, Fuzhou National Forest Park; verbatimElevation: 193.5; verbatimLatitude: 26°10′27.3″N; verbatimLongitude: 119°16′25.8″E; **Event:** samplingProtocol: by hand; year: 2024; month: 1; day: 29; **Record Level:** institutionCode: FJFZ-24-10-01

#### Description

**Figure 1.**
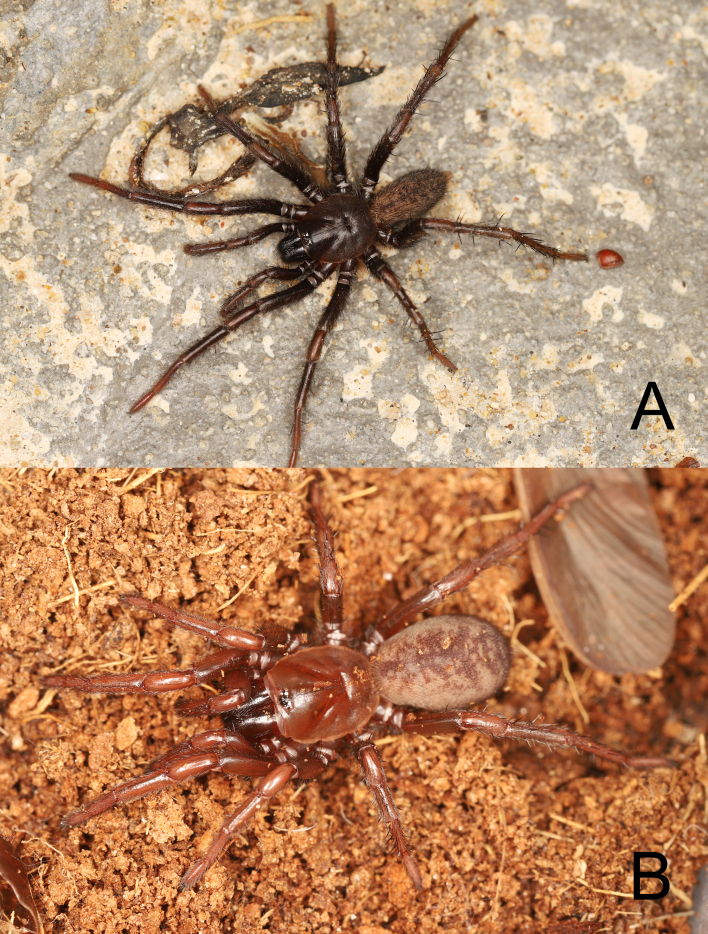
Photos of live specimens of *Raveniolafuzhouensis* Zhou, sp. nov. **A** male (holotype); **B** female (paratype).

**Figure 2.**
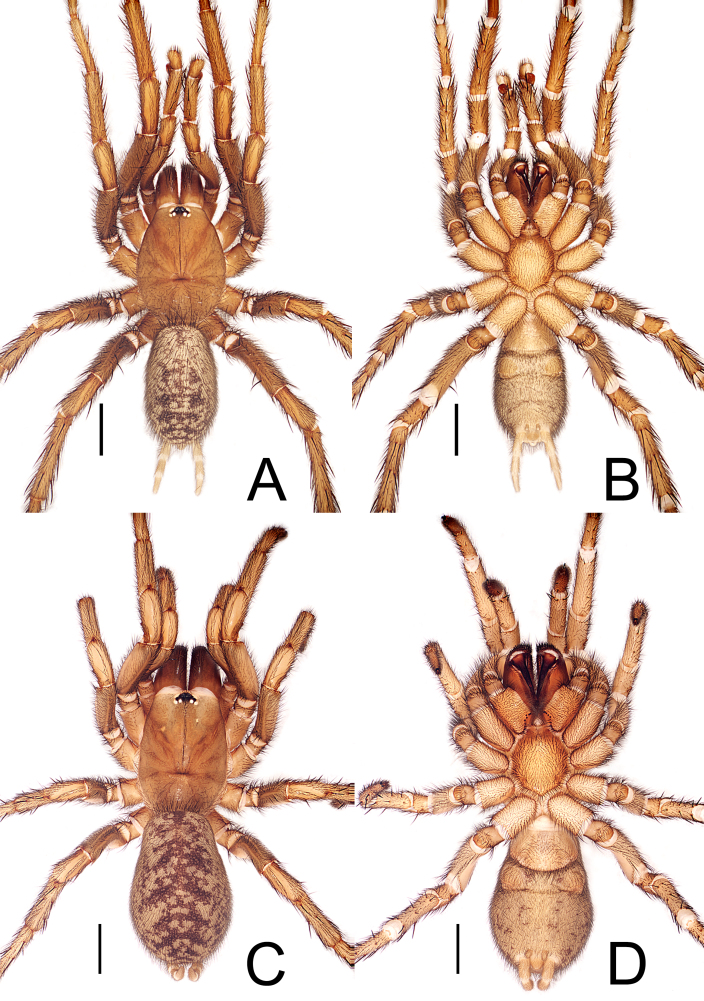
*Raveniolafuzhouensis* Zhou, sp. nov., habitus. **A, B** male (holotype), **C, D** female (paratype). **A, C** dorsal view; **B, D** ventral view. Scale bars: 2 mm (A-D).

**Figure 3.**
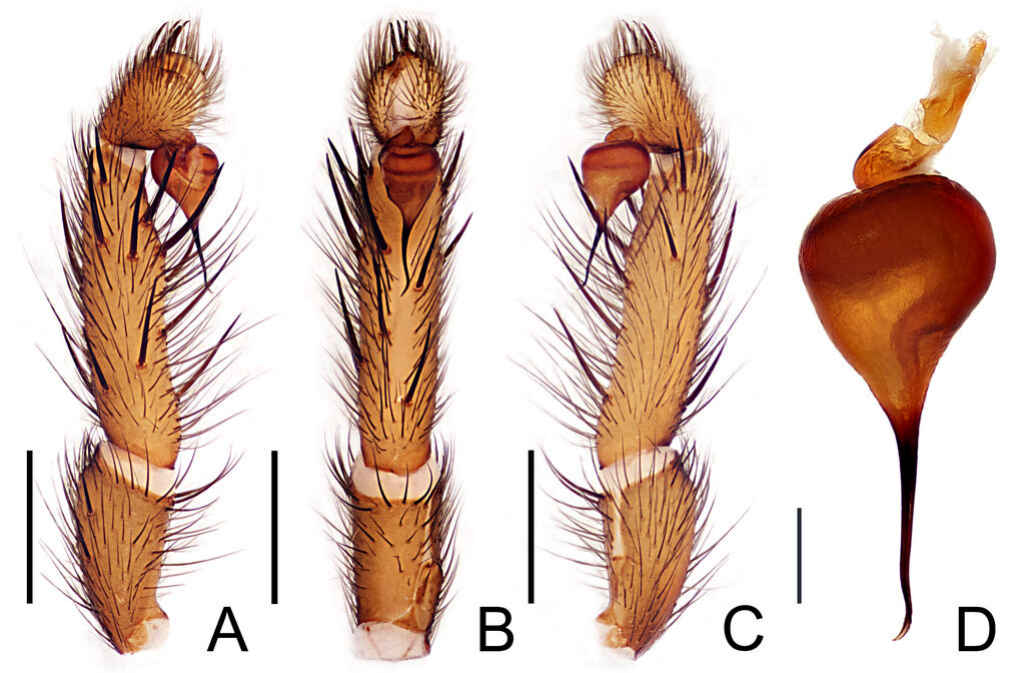
*Raveniolafuzhouensis* Zhou, sp. nov., holotype male. **A** left palp, prolateral view; **B** same, ventral view; **C** same, retrolateral view; **D** palpal bulb, retrolateral view. Scale bars: 1 mm (A–C); 0.2 mm (D).

**Figure 4.**
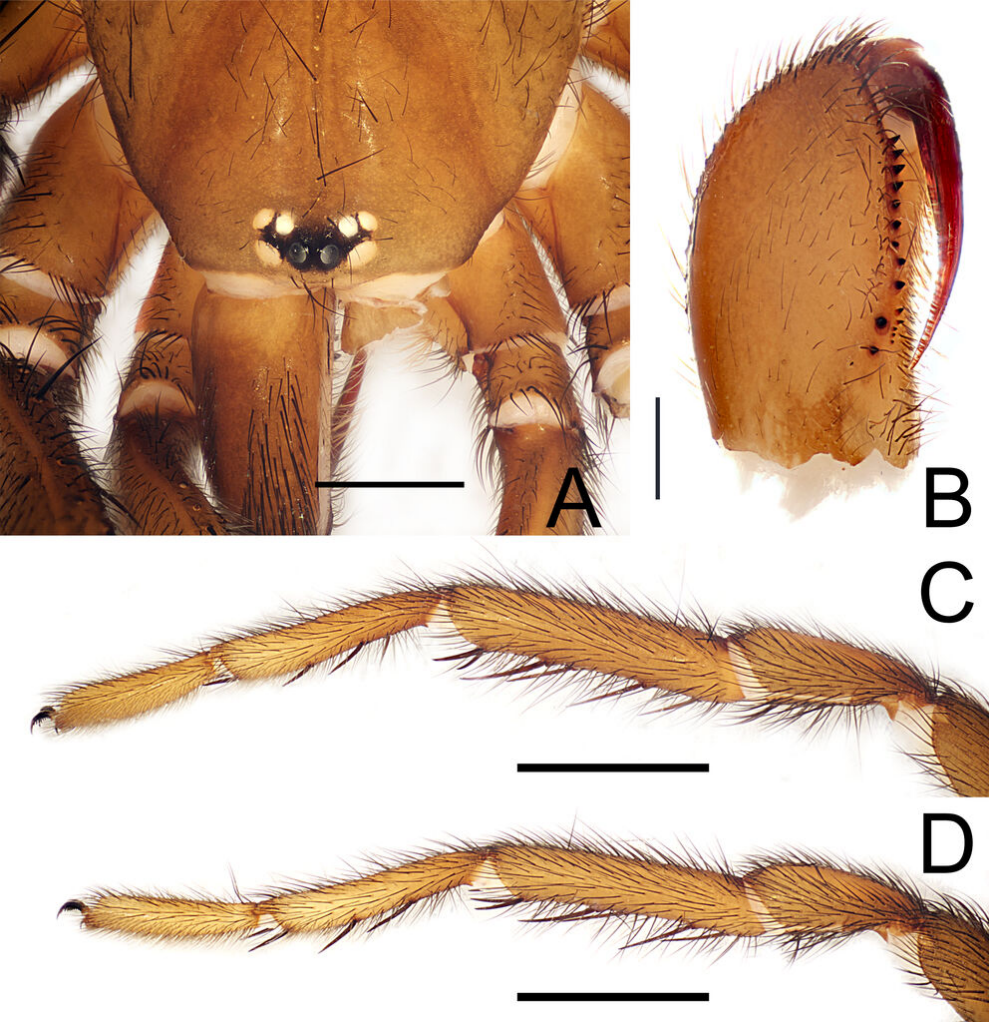
*Raveniolafuzhouensis* Zhou, sp. nov., holotype male. **A** front part of prosoma, dorsal view; **B** chelicerae, pro-ventral view; **C** leg I, retrolateral view; **D** leg II, retrolateral view. Scale bars: 1 mm (A); 0.5 mm (B); 2 mm (C-D).

**Male** (holotype; FJFZ-24-19-03,Fig. 1A and Fig. 2A). TL 9.76 (not included chelicerae), CL 4.85, CW 3.62, AL 5.02, AW 3.04. Eye sizes and distances: AME 0.14, ALE 0.22, PME 0.16, PLE 0.18, AME–AME 0.08, AME–ALE 0.05, PME–PME 0.28, PME–PLE 0.02. Leg lengths: leg I: 13.68 (3.92, 2.15, 3.15, 2.54, 1.92), leg II: 12.83 (3.69, 1.92, 2.85, 2.52, 1.85), leg III: 11.76 (3.23, 1.38, 2.46, 2.92, 1.77), leg IV: 16.31 (4.24, 1.85, 3.54, 4.46, 2.23). Leg formula: 4123. Carapace yellowish-brown dorsally, with short black setae. Eye tubercle middle blackish-brown, AME black, other eyes white (Fig. 4A). Chelicerae 1.62 long, dark brown, each cheliceral furrow with 11 promarginal teeth and 5 mesobasal denticles (Fig. 4B). Maxillae 2.54 long and 1.45 wide, each with 4 cuspules beside a ridge (Fig. 2B). Labium 0.61 long, 0.73 wide; sternum 2.62 long and 2.08 wide, with one pair of suborbicular sigilla (measurements of sigilla: lower = 0.21) (Fig. 2B). Sternum, labium, maxillae and legs greyish-brown ventrally. Abdomen dorsally light brown, with blackish cloudy maculae and black setae (Fig. 2A). Ventral surface of abdomen and spinnerets brown, with dense black setae (Fig. 2B). Chelicerae dorsally front end with strong black setae (Fig. 1A and Fig. 4A). Spinnerets: PMS length 0.24, diameter 0.11. PLS maximal diameter 0.39; length of basal, medial and apical segments 1.02, 0.68, 0.71; total length 2.41; apical segment triangular (Fig. 2A, B). Spination. Palp: femur d4, p1; patella p2; tibia pd3, rd3, v5, pv2, r2; cymbium d4. Leg I: femur d4, pd2; patella aspinose; tibia p2, rv3+4M; metatarsus v5, p1. Leg II: femur d4, pd2; patella p1; tibia p2, v7; metatarsus v6. Leg III: femur d3, pd3, rd2; patella p3, rd2, v7; tibia d2, pd3, rd2, v7; metatarsus pd4, rd2, v7. Leg IV: femur d3, pd3, rd2; patella pd1, rd1; tibia d2, pd3, rd2, v7; metatarsus pd6, rd6, v8. Tarsi I–IV aspinose. Tibia, cymbium and copulatory bulb of palp as shown in Fig. 3 and Fig. 7A-D.

Palpal tibia scattered 11 thick and long spines; cymbium with five stout spines; embolus base globose, brownish-brown, middle slender outstretched, slender tubular and black, its end slightly bent and tapering (Fig. 3A-D and Fig. 7A-D).

**Female** (paratype; FJFZ-24-19-04, Fig. 1B and Fig. 2C): TL 11.62 (not included chelicerae), CL 5.23, CW 3.85, AL 6.46, AW 4.38. Eye sizes and interdistances: AME 0.16, ALE 0.29, PME 0.16, PLE 0.18, AME–AME 0.11, AME–ALE 0.04, PME–PME 0.31, PME–PLE 0.29. Leg lengths: leg I: 10.84 (3.92, 2.15, 3.15, 2.54, 1.92), leg II: 12.83 (3.69, 1.92, 2.85, 2.52, 1.85), leg III: 11.76 (3.23, 1.38, 2.46, 2.92, 1.77), leg IV: 16.31 (4.24, 1.85, 3.54, 4.46, 2.23). Leg formula: 4123. Carapace grey-brown dorsally, with a few black setae. Eye tubercle middle blackish-brown, AME black, other eyes white (Fig. 6A). Chelicerae 1.62 long, dark brown, each cheliceral furrow with 12 promarginal teeth and 6-7 mesobasal denticles (Fig. 6C). Maxillae 2.25 long and 1.35 wide, each with 5 cuspules beside a ridge (Fig. 6A). Labium 0.41 long, 0.58 wide; sternum 3.02 long and 2.38 wide, with three pairs of suborbicular sigilla (measurements of sigilla: upper: median: lower = 0.10: 0.11: 0.22) (Fig. 6A). Sternum, labium, maxillae and legs dark brown ventrally. PMS length 0.42, diameter 0.21. PLS maximal diameter 0.44; length of basal, medial and apical segments 0.82, 0.72, 0.56; total length 2.10; apical segment triangular (Fig. 2C and D). Spination. Palp: femur d1; patella aspinose; tibia v6; tarsi v4. Leg I and II: femur p1; patella aspinose; tibia v3; metatarsus v5, p1. Leg III: femur pd1; patella pd1, rd2; tibia d2, pv2, pd2, rd2, v6; metatarsus d4, pd3, rd4, v8. Leg IV: femur pd1; patella pd1, rd1; tibia d2, pd3, rd2, pv2, rv2, v6; metatarsus pd2, d6, rd2, v8. Tarsi I–IV aspinose.

**Figure 5.**
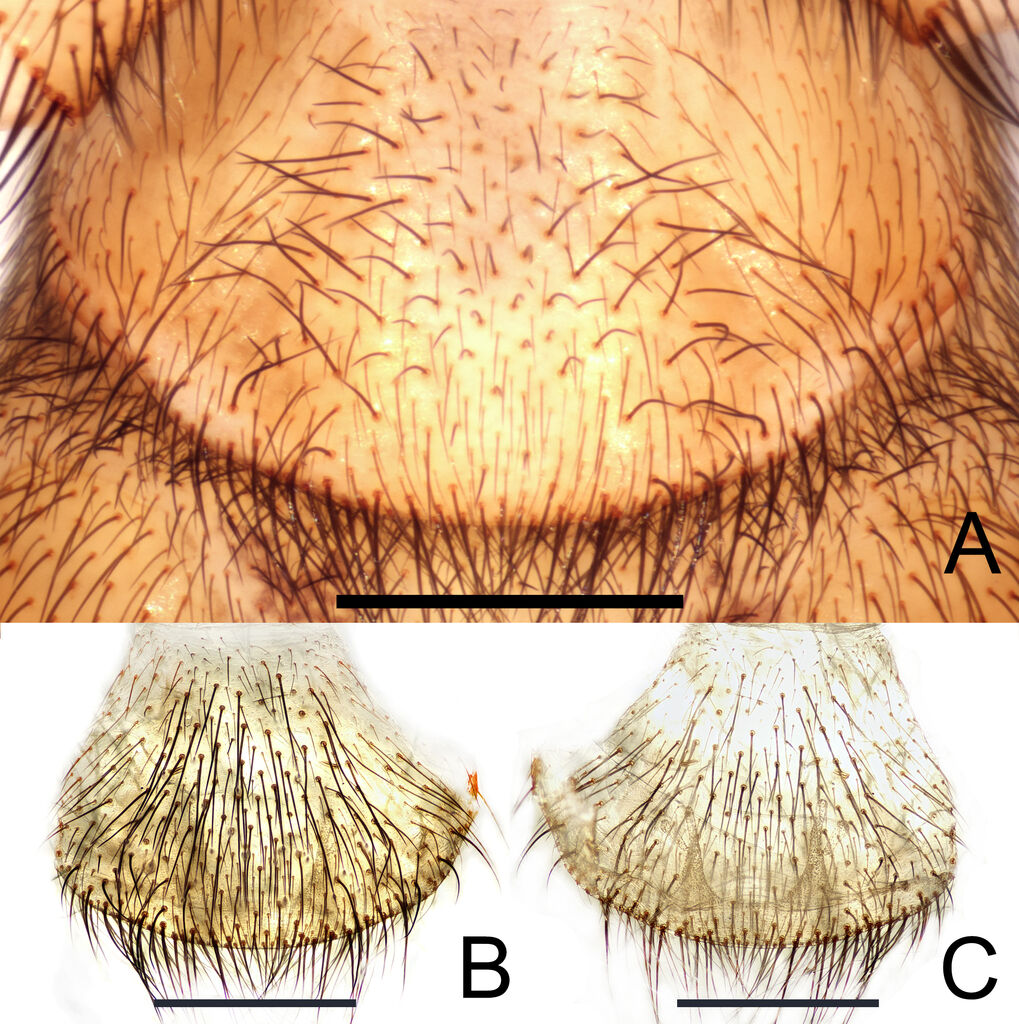
*Raveniolafuzhouensis* Zhou, sp. nov., paratype female. **A, B** epigyne, ventral view; **C** vulva, dorsal view. Scale bars: 1 mm (A-C).

**Figure 6.**
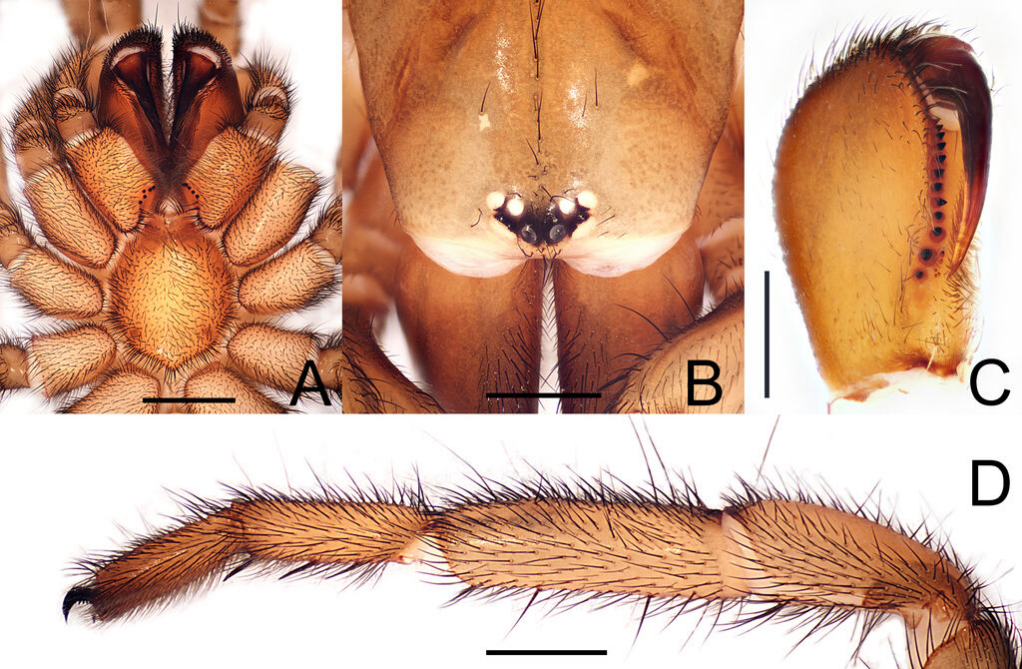
*Raveniolafuzhouensis* Zhou, sp. nov., paratype female. **A** labium and sternum of prosoma, ventral view; **B** front part of prosoma , dorsal view; **C** chelicerae, pro-ventral view; **D** leg I, retrolateral view. Scale bars: 2 mm (A); 1 mm (B-D).

**Figure 7.**
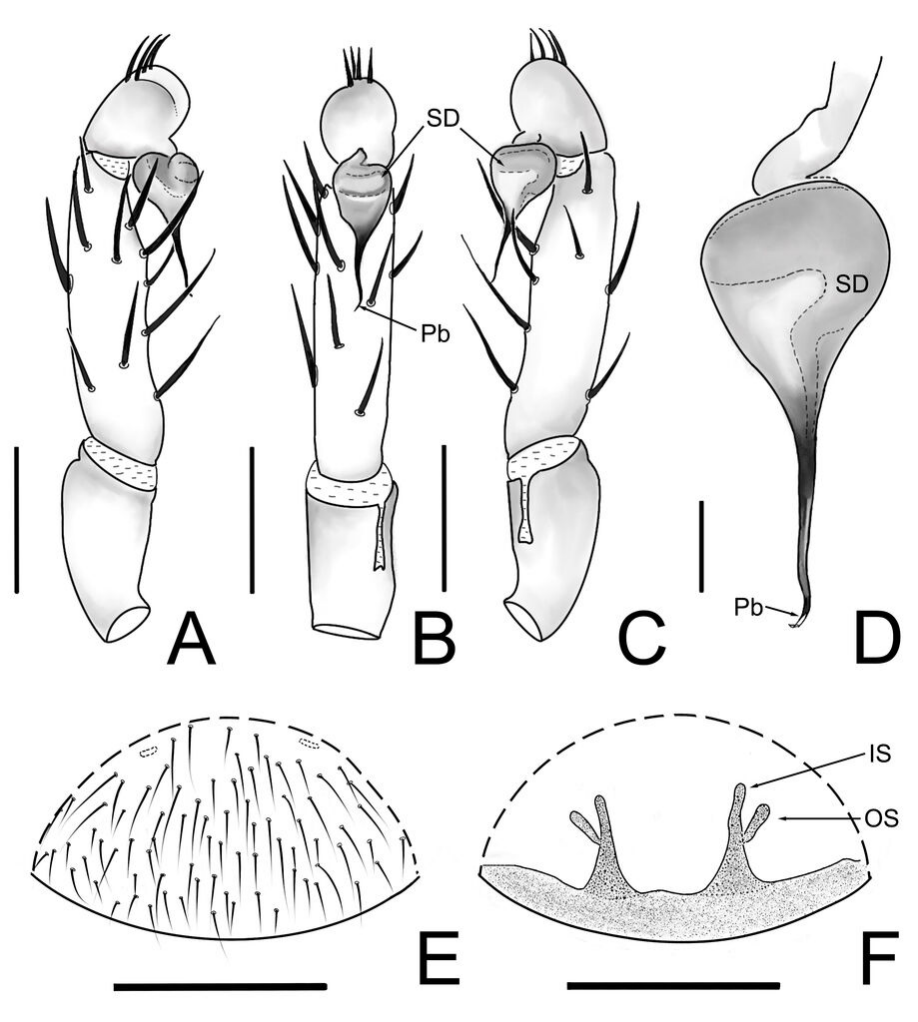
*Raveniolafuzhouensis* Zhou, sp. nov., **A-D** holotype male, **E-F** paratype female. **A-C** left palp; **D** palpal bulb; **E** epigyne; **F** vulva. **A** prolateral view; **B, E** ventral view; **C-D** retrolateral view; **F** dorsal view. Abbreviations: IS = inner spermathecal branch, OS = outer spermathecal branch, Pb = psembolus, SD = sperm duct. Scale bars: 1 mm (A–C, E-F); 0.2 mm (D).

Spermathecae white, narrow and bifurcated, width and length of the bifurcation part are similar (Fig. 5C and Fig. 7F), distance between spermathecae 0.26, IS length 0.23/0.27, OS length 0.2/0.21, spermathecae base width 0.28/0.33, stalk of spermathecae trumpet-shaped and height 0.26. Dense pores are scattered on the surface of spermathecae.

##### Variation

Tolal length males (n = 7) varies from 10.64 to 12.07 (included chelicerae). Carapace length in males (n = 7) varies from 4.71 to 5.57.

#### Diagnosis

The new species is similar to *Raveniolagracilis* Li & Zonstein, 2015 (Tian et al. 2020: figs. 1C-D, 2, 3C-D and Li and Zonstein 2015: figs. 9A-C and 10) in the male and female individual abdomen back pattern approximation. The male of the new species can be easily distinguished from *R.gracilis* by the following: (1) *embolus* base wider and black (Fig. 3) vs. embolus of *R.gracilis* dark brown (Li and Zonstein 2015: fig. 9; Tian et al. 2020: fig. 3D); (2) SD base the pipeline smooth down to embolus (Fig. 3B) vs. SD base the pipeline 90° to embolus of *R.gracilis* (Li and Zonstein 2015: figs. 9B and 10B). The female of the new species can be easily distinguished from *R.gracilis* by the following: (1) the stalk of the spermathecae is significantly wider (trumpet-shaped), with the basal width approximately same as that of the IS, gradually narrowing upwards to the junction of the IS and OS; (2) the OS is straight, emerging obliquely from the stalk to form an angle of about 40° with the IS (in contrast, in *R.gracilis*, the base of the stalk of the spermathecae does not significantly widen, with a width close to that of the IS or up to twice the width of the IS; the OS emerges perpendicularly or downwards from the stalk, then extends upwards, forming a curved structure near the base, see Tian et al. (2020): fig. 2).

#### Etymology

The specific name refers to the type locality, adjective.

#### Distribution

**Figure 8.**
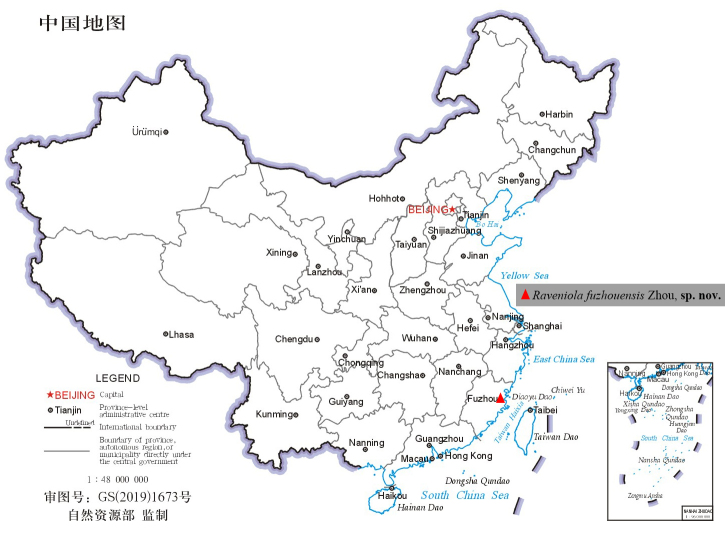
Distribution record of *Raveniolafuzhouensis* Zhou, sp. nov.

China (Known only from type locality in Fujian; Fig. 8).

#### Biology

*R.fuzhouensis* Zhou, sp. nov. lives in dry soil burrows on loess road cuts or cavities beneath flat stones. The excavation marks inside the burrow indicate their ability to further modify the burrow, rather than passively adapting to the existing environment.

## Supplementary Material

XML Treatment for Raveniola fuzhouensis
